# Simulation Methods to Inform Study Design and Understand Potential Bias: The Example of Random Error in AD Biomarkers

**DOI:** 10.1093/geroni/igaf122.1856

**Published:** 2025-12-31

**Authors:** Eleanor Hayes-Larson, Sarah Ackley

**Affiliations:** University of Southern California, Los Angeles, California, United States; Brown University, Providence, Rhode Island, United States

## Abstract

Novel blood-based biomarkers for Alzheimer’s disease pathology are increasingly available and are appealing options for inclusion in population-based studies due to numerous advantages related to cost and low invasiveness. However, they are subject to more random measurement error (i.e., noise) relative to more traditional biological measures such as neuroimaging or CSF. We leverage data from the Alzheimer’s Disease Neuroimaging Initiative and use a simulation study to highlight potential implications of random measurement error for power and sample size requirements when using blood-based biomarkers (e.g., p-tau 181) vs. neuroimaging (amyloid PET) to understand the effects of risk factors like age on biomarker levels, and additional implications for bias when using the biomarker as an independent variable to evaluate the relationship between biomarkers and cognitive outcomes (e.g. Clinical Dementia Rating Sum of Boxes). More broadly, we highlight the benefits of simulation studies to inform study design, understand sources of bias, and investigate other methodological issues in population aging research.

